# Patient Profile and Diagnostic Distribution in a Turkish Rheumatology Outpatient Clinic: A Prospective 2-Month Analysis

**DOI:** 10.5152/eurasianjmed.2026.261504

**Published:** 2026-08-24

**Authors:** Selin Cilli Hayıroğlu, Ender İğneci

**Affiliations:** Department of Rheumatology, İstanbul Göztepe Prof. Dr. Süleyman Yalçın City Hospital, İstanbul, Türkiye

**Keywords:** Rheumatic diseases, outpatients, ambulatory care, cross-sectional studies, referral and consultation

## Abstract

**Background:**

This study aimed to evaluate the clinical and demographic profile of patients attending a rheumatology outpatient clinic in Türkiye over a 2-month period, with a focus on symptom distribution, visit types, and rheumatologic diagnostic outcomes.

**Methods:**

This prospective cross-sectional study was conducted at a rheumatology outpatient clinic in Türkiye. A total of 971 patients were included over a 2-month period. Patients were categorized as follow-up (n = 491), newly diagnosed (n = 45), or other/first visit (n = 435). Demographic data, presenting symptoms, laboratory findings, and final diagnoses were recorded.

**Results:**

The mean age of the cohort was 51.3 years (median: 53 years), and 74.0% were female (n = 719). Among the 480 patients without a prior rheumatologic diagnosis, the most common presenting symptom was peripheral joint pain (55.8%), followed by low back pain (7.9%) and myalgia (7.5%). Of these, 45 (9.4%) received a new rheumatologic diagnosis. Overall, 55.2% of all patients (n = 536) carried a rheumatologic diagnosis. Among patients with a rheumatologic diagnosis, the most frequent were rheumatoid arthritis (30.4%), ankylosing spondylitis (13.9%), and Sjögren syndrome (8.9%). Compared to patients without a diagnosis, newly diagnosed patients had significantly higher rates of myalgia, arthritis, elevated acute phase reactants, Raynaud’s phenomenon, and abdominal pain (*P* < .05).

**Conclusion:**

This study provides a comprehensive snapshot of the rheumatology outpatient population in Türkiye. Peripheral joint pain is the predominant presenting complaint, and over half of patients carry an established rheumatologic diagnosis. Identifying symptom patterns associated with new diagnoses may facilitate earlier recognition and referral in clinical practice.

**Main Points**

- Over half of all patients attending a tertiary rheumatology outpatient clinic in Türkiye carried an established rheumatologic diagnosis, with follow-up visits accounting for the majority of clinic activity, highlighting the significant burden of chronic disease management on subspecialty resources.
- Peripheral joint pain was the most common presenting complaint (21.8%), followed by back pain (10.9%) and myalgia (7.8%), reflecting the broad spectrum of musculoskeletal conditions referred to rheumatology services.
- Rheumatoid arthritis (30.4%), ankylosing spondylitis (13.9%), and familial Mediterranean fever (13.0%) were the most frequently encountered diagnoses, with Sjögren syndrome (8.9%) representing the most common connective tissue disease in the cohort.
- Newly diagnosed patients were significantly more likely to present with objective inflammatory features—including arthritis, elevated acute phase reactants, Raynaud's phenomenon, and abdominal pain—compared to undiagnosed patients, suggesting that these findings may be useful in guiding triage decisions; however, prospective validation in larger, multicentre studies is needed before formal referral recommendations can be made.
- The low rheumatologist density in Türkiye (approximately 0.22 per 100000 population) underscores the urgent need for structured referral criteria, enhanced primary care education, and innovative care delivery models to optimize the use of limited subspecialty resources.

## Introduction

Rheumatology is an internal medicine subspecialty that encompasses a broad spectrum of inflammatory, autoimmune, and musculoskeletal disorders. As a relatively young and rapidly evolving field, rheumatology clinics serve a heterogeneous patient population ranging from those with well-established diagnoses requiring long-term follow-up to individuals presenting with undifferentiated symptoms that may or may not represent a systemic rheumatic disease. Understanding the composition of this patient population is essential not only for optimizing clinical workflows but also for informing healthcare planning, training priorities, and resource allocation.

In Türkiye, rheumatology has developed significantly over the past 2 decades; however, the number of practicing rheumatologists remains limited relative to the population size. As reported by Arıcan and Tezcan, there are approximately 0.22 rheumatologists per 100000 persons in Türkiye, a figure that underscores the substantial unmet need for subspecialty care in the country.^1^ This scarcity of specialists places considerable pressure on existing rheumatology outpatient clinics, which must balance the demands of chronic disease management with the evaluation of new and undifferentiated referrals. Globally, the rheumatology workforce shortage is a recognized challenge; projections suggest that demand for rheumatologic care will outstrip supply by over 100% by 2030 in many regions, driven by an aging population, increasing prevalence of autoimmune diseases, and expanding therapeutic options that require specialist oversight.^2^

Against this backdrop, characterizing the patient profile of a rheumatology outpatient clinic provides valuable insight into how subspecialty resources are being utilized and where improvements in referral pathways, triage systems, and primary care education may be warranted. Several studies from Türkiye and other countries have examined the spectrum of diagnoses and referral patterns in rheumatology settings. Arıcan and Tezcan reported that 53.1% of patients referred to a rheumatology clinic received a rheumatologic diagnosis, with articular signs being the most common reason for referral.^1^ Similarly, an important investigation demonstrated that among patients referred from general practice, the threshold for referral was relatively high, with long delays before subspecialty consultation.^3^ These findings highlight the importance of understanding local referral dynamics and patient characteristics to improve the efficiency and quality of rheumatologic care.^4^

The present study was conducted at a tertiary rheumatology outpatient clinic in İstanbul, Türkiye, over a 2-month period. The primary aim was to describe the demographic and clinical profile of all patients attending the clinic, including both follow-up and new referrals. A secondary aim was to identify clinical and laboratory features that distinguish patients who receive a new rheumatologic diagnosis from those who remain undiagnosed, with the broader goal of informing strategies to optimize the use of rheumatology subspecialty resources.

## Material and Methods

This prospective cross-sectional study was conducted between January 2026 and March 2026 at the Department of Rheumatology, Istanbul Göztepe Prof. Dr. Süleyman Yalçın City Hospital, a tertiary-care university-affiliated hospital serving a large urban population in Istanbul, Türkiye. The study was designed and reported in accordance with the Strengthening the Reporting of Observational Studies in Epidemiology (STROBE) guidelines for cross-sectional studies. The study was conducted in accordance with the principles of the Declaration of Helsinki. Ethical approval was obtained from Medeniyet University Faculty of Medicine Ethics Committee (Approval number: 328, Approval date: 18.12.2025). Written informed consent was obtained from all participants prior to enrollment. This approach was approved by Medeniyet University Faculty of Medicine Research Ethics Committee. The 2-month enrollment window was selected to provide a representative cross-sectional snapshot of clinical activity and was considered sufficient to achieve a robust sample size for describing patient profiles and diagnostic distribution, rather than assessing longitudinal treatment outcomes.

All patients aged 18 years or older who attended the rheumatology outpatient clinic during the study period were consecutively enrolled. No patients were excluded based on diagnosis, referral source, or visit frequency; the only exclusion criterion was age under 18 years. Patients were categorized into three groups based on their visit type: (1) follow-up patients with a pre-existing rheumatologic diagnosis, (2) newly diagnosed patients who received a new rheumatologic diagnosis during the study period, and (3) other/first-visit patients who presented for the first time without a prior rheumatologic diagnosis and did not receive a new diagnosis during the study period. Diagnoses were established by the attending rheumatologist based on current internationally recognized classification or diagnostic criteria applicable to each condition. Specifically, rheumatoid arthritis (RA) was classified according to the 2010 ACR/EULAR criteria; ankylosing spondylitis (AS) according to the modified New York criteria; psoriatic arthritis (PsA) according to the CASPAR criteria; axial spondyloarthritis (axSpA) according to the 2009 ASAS criteria; familial Mediterranean fever (FMF) according to the Tel Hashomer criteria; Behçet’s disease according to the International Study Group criteria; gout according to the 2015 ACR/EULAR classification criteria; Sjögren’s syndrome according to the 2016 ACR/EULAR criteria; systemic lupus erythematosus (SLE) according to the 2019 EULAR/ACR criteria; and systemic sclerosis (SSc) according to the 2013 ACR/EULAR criteria.

For each patient, the following data were recorded: age, sex, presenting symptoms and findings, relevant laboratory parameters (including C-reactive protein [CRP], erythrocyte sedimentation rate [ESR], rheumatoid factor [RF], antinuclear antibody [ANA]), and final rheumatologic diagnosis where applicable.

The following operational definitions were applied consistently throughout the study. Elevated acute phase reactants (APR) were defined as CRP > 5 mg/L and/or ESR exceeding the age- and sex-adjusted upper limit of normal (> 20 mm/h in men aged < 50 years, > 30 mm/h in men aged ≥ 50 years, > 30 mm/h in women aged < 50 years, and > 40 mm/h in women aged ≥5 0 years), in accordance with standard laboratory reference ranges. RF positivity was defined as a serum RF titer ≥ 14 IU/mL as determined by nephelometry, in accordance with the local laboratory reference range. ANA positivity was determined by indirect immunofluorescence (IIF) on HEp-2 cells; a titer of ≥ 1:80 was considered positive, consistent with current ACR/EULAR recommendations. Arthritis was defined as the presence of objective joint swelling with or without warmth and tenderness on physical examination, as documented by the attending rheumatologist. Dry mouth was recorded when the patient reported persistent oral dryness, with or without objective evidence of salivary gland dysfunction. Raynaud’s phenomenon was defined as episodic, well-demarcated color changes of the digits (pallor, cyanosis, and/or erythema) triggered by cold or emotional stress, as reported by the patient and/or documented by the clinician. Ocular pathology was recorded when the patient presented with or was referred for uveitis, episcleritis, scleritis, or sicca-related ocular symptoms (dry eye), as documented in the clinical notes or ophthalmology referral. Skin findings were recorded when the patient presented with clinician-documented skin redness, rash, or other dermatological manifestations potentially attributable to a systemic rheumatic disease. Presenting symptoms were categorized based on the primary complaint documented at the time of the visit.

Data were collected prospectively using a structured data form completed at the time of each visit. To ensure data accuracy, a dual verification approach was employed: clinical information was cross-referenced between the referring physician’s notes—including referral letters and outpatient records retrieved from the hospital information system—and the patient’s self-reported presenting complaints recorded during the visit. Any discrepancies between the 2 sources were resolved by direct review of the clinical encounter documentation. To minimize selection bias, all consecutive attendees during the study period were included without pre-selection. As data were collected prospectively at the point of care, recall bias was not applicable. No patients were lost to follow-up within the study period, as data capture was completed at the time of each clinic visit.

### Statistical Analysis

As this was a descriptive cross-sectional study with a fixed 2-month enrollment window, no formal sample size calculation was performed. The study population comprised all consecutive patients attending the clinic during the study period, and the resulting sample size (n = 971) was considered adequate for the descriptive and comparative objectives of the study.

Continuous variables were tested for normality using the Kolmogorov–Smirnov test and are presented as median [interquartile range (IQR)] due to non-normal distribution. Categorical variables are expressed as frequency (n) and percentage (%). Group comparisons were performed using the Mann–Whitney *U-*test for continuous variables, and the chi-square or Fisher’s exact test for categorical variables, as appropriate. All statistical analyses were performed using SPSS version [26.0] (IBM Corp., Armonk, NY, USA). A *P*value of < 0.05 was considered statistically significant. Because only 45 patients received a new rheumatologic diagnosis and several candidate predictors had sparse cell counts, a conventional multivariable logistic regression model was not performed due to the risk of overfitting and unstable estimates.

## Results

A total of 971 patients were included in the study. The median age of the cohort was 53 years [IQR: 41-62], with a mean age of 51.3 years. The majority of patients were female (n = 719, 74.0%), while 252 (26.0%) were male. The most represented age group was 45-59 years (n = 343, 35.3%), followed by 60-74 years (n = 276, 28.4%) and 30-44 years (n = 208, 21.4%). Patients aged 75 years and older constituted 4.6% of the cohort (n = 45), and those aged 18-29 years accounted for 10.2% (n = 99). Demographic characteristics are summarized in Table 1.

### Visit Type Distribution

**Figure 1.**
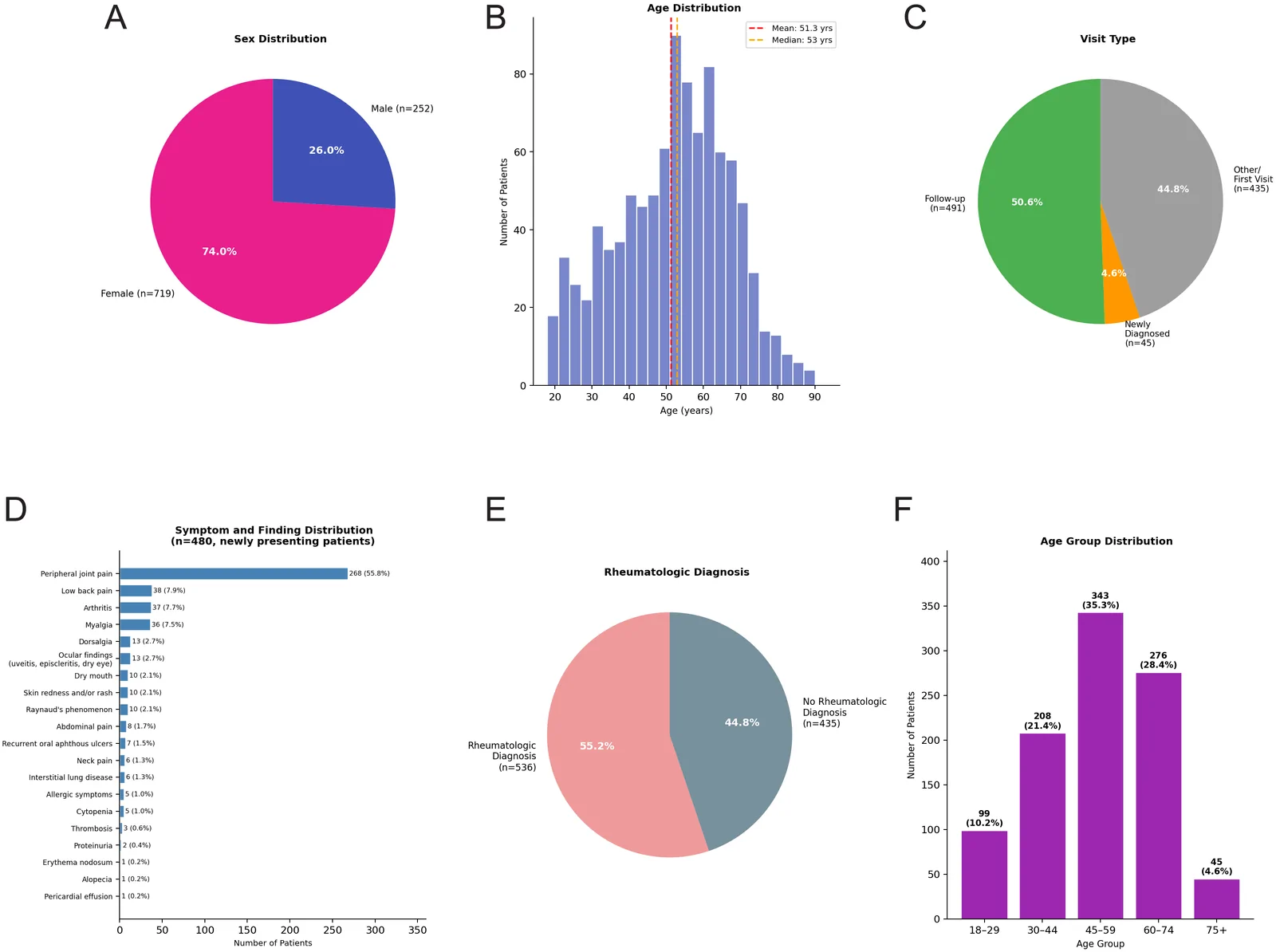
Demographic and Clinical Profile of the Rheumatology Outpatient Cohort (n = 971).(A) Sex distribution showing a female predominance (74.0%). (B) Age distribution of the total population with a mean age of 51.3 years and median of 53 years. (C) Distribution of patients by visit type: Follow-up visits (50.6%), Other/First visits (44.8%), and Newly diagnosed cases (4.6%). (D) Distribution of presenting symptoms and clinical findings among newly presenting patients (n = 480), highlighting peripheral joint pain as the leading complaint. (E) Distribution of final diagnostic outcomes, where 55.2% of the cohort carried a rheumatologic diagnosis. (F) Age group distribution, showing the highest patient density in the 45-59 years age category.

Of the 971 patients, 491 (50.6%) were follow-up patients with established rheumatologic diagnoses, 435 (44.8%) were other/first-visit patients without a prior or new rheumatologic diagnosis, and 45 (4.6%) received a new rheumatologic diagnosis during the study period (Figure 1C). Overall, 536 patients (55.2%) carried a rheumatologic diagnosis at the end of the study period (Figure 1E).

### Symptom Distribution

The most frequently reported presenting symptom among the 480 patients without a prior rheumatologic diagnosis (undiagnosed and newly diagnosed patients) was peripheral joint pain (n = 268, 55.8%), followed by low back pain (n = 38, 7.9%), arthritis (n = 37, 7.7%), and myalgia (n = 36, 7.5%). Less common presentations included dorsalgia (n = 13, 2.7%), ocular findings such as uveitis, episcleritis, and dry eye (n = 13, 2.7%), dry mouth (n = 10, 2.1%), skin redness and/or rash (n = 10, 2.1%), Raynaud’s phenomenon (n = 10, 2.1%), abdominal pain (n = 8, 1.7%), recurrent oral aphthous ulcers (n = 7, 1.5%), neck pain (n = 6, 1.3%), interstitial lung disease (n = 6, 1.3%), allergic symptoms (n = 5, 1.0%), cytopenia (n = 5, 1.0%), thrombosis (n = 3, 0.6%), proteinuria (n = 2, 0.4%), erythema nodosum (n = 1, 0.2%), alopecia (n = 1, 0.2%), and pericardial effusion (n = 1, 0.2%). The full symptom distribution is presented in Table 2 and Figure 1D.

### Rheumatologic Diagnoses

Among the 536 patients with a rheumatologic diagnosis, the most common diagnostic category was inflammatory arthritis (n = 291, 54.2%), with rheumatoid arthritis (RA) being the most frequent individual diagnosis (n = 163, 30.4%), followed by ankylosing spondylitis (AS) (n = 75, 13.9%) and psoriatic arthritis (PsA) (n = 37, 6.9%). Juvenile idiopathic arthritis (JIA) was identified in 2 patients (0.3%). Among autoinflammatory and crystal arthropathies, familial Mediterranean fever (FMF) was the most prevalent (n = 70, 13.0%), followed by gout (n = 45, 8.4%), Behçet disease (n = 23, 4.3%), and pseudogout (n = 4, 0.7%). Among connective tissue diseases, Sjögren syndrome was the most common (n = 48, 8.9%), followed by SLE (n = 13, 2.4%), undifferentiated CTD (n = 10, 1.8%), and systemic sclerosis (SSc) (n = 9, 1.7%). The complete diagnostic distribution is presented in Table 3.

### Comparison of Newly Diagnosed vs. Undiagnosed Patients

Among the 480 newly presenting patients, 45 (9.4%) received a new rheumatologic diagnosis during the study period. Newly diagnosed patients (n = 45) and undiagnosed patients (n = 435) did not differ significantly in terms of age (median 51 [IQR: 42-60] vs. 53 [IQR: 40-62], *P* = .639) or sex distribution (female: 82.2% vs. 71.5%, *P* = .174). However, several clinical features were significantly more prevalent in the newly diagnosed group. Peripheral joint pain (73.3% vs. 54.0%, *P* = .020), myalgia (40.0% vs. 4.1%, *P* < .001), arthritis (42.2% vs. 4.1%, *P* < .001), elevated APR (24.4% vs. 3.0%, *P* < .001), dry mouth (11.1% vs. 1.1%, *P* < .001), Raynaud’s phenomenon (20.0% vs. 0.2%, *P*< 0.001), abdominal pain (13.3% vs. 0.5%, *P* < 0.001), and cytopenia (8.9% vs. 0.2%, *P* < .001) were significantly more common in the newly diagnosed group. Back pain was also significantly more common in the newly diagnosed group (17.8% vs. 6.9%, *P* = .022). Ocular pathology was significantly more prevalent in newly diagnosed patients (13.3% vs. 1.6%, *P* < .001). Arterial/venous thrombosis was not observed in the newly diagnosed group (0.0% vs. 0.9%, *P* = 1.000). Proteinuria (2.2% vs. 0.2%, *P* = .179) and interstitial lung disease (4.4% vs. 0.9%, *P* = .186) did not reach statistical significance between groups. Detailed comparisons are presented in Table 4.

## Discussion

This prospective study provides a comprehensive characterization of the patient population attending a tertiary rheumatology outpatient clinic in İstanbul, Türkiye, over a 2-month period. The findings offer important insights into the utilization patterns of rheumatology subspecialty services and the clinical features that distinguish patients who receive a new rheumatologic diagnosis from those who remain undiagnosed.

The predominance of female patients (74.0%) in the cohort is consistent with the well-established epidemiological pattern of autoimmune and inflammatory rheumatic diseases, which disproportionately affect women.^5^ The median age of 53 years reflects the typical age distribution of rheumatic disease onset and the chronic nature of these conditions, which necessitate long-term specialist follow-up. The relatively small proportion of patients aged 75 years and older (4.6%) may reflect barriers to access in elderly populations or the tendency for older patients with established diagnoses to be managed in primary care settings.

A striking finding of this study is that over half of all clinic visits (50.6%) were accounted for by follow-up patients with established diagnoses. This pattern reflects the chronic and relapsing nature of rheumatic diseases, which require ongoing specialist monitoring, treatment adjustment, and laboratory surveillance. The high proportion of follow-up visits has important implications for clinic capacity and resource planning.^6^ In a setting with limited rheumatologist availability in Türkiye, this burden may restrict access for new referrals and delay diagnosis.^1^This imbalance between follow-up care and new patient access has also been reported globally and highlights the need for more efficient care models.^7^

Peripheral joint pain was by far the most common presenting complaint (55.8%, n = 268/480), followed by back pain (7.9%) and myalgia (7.5%). An important study similarly identified articular signs as the most common reason for referral to a rheumatology clinic in Türkiye, with 53.1% of referred patients ultimately receiving a rheumatologic diagnosis—a figure comparable to the overall diagnostic yield of 55.2%.^1^ The high prevalence of musculoskeletal complaints as the primary referral indication reflects both the broad differential diagnosis of joint and soft tissue pain and the limited capacity of primary care providers to differentiate inflammatory from non-inflammatory musculoskeletal conditions. This highlights the need for improved referral accuracy in primary care, as emphasized by recent investigations, which noted that inappropriate use of laboratory tests and long delays before referral were common in general practice.^3^ The diagnostic spectrum observed in the cohort reflects the full breadth of rheumatologic practice. Rheumatoid arthritis was the most common individual diagnosis (30.4%), consistent with its estimated prevalence of approximately 0.5-1.0% in the Turkish population. The notable proportion of FMF (13.0%) is consistent with the higher prevalence of autoinflammatory diseases in the Turkish population, reflecting the well-documented geographic clustering of FMF along the ancient Silk Road. Gout (8.4%) and Behçet disease (4.3%) were also prominent, the latter being particularly relevant in the Turkish context given the higher prevalence of Behçet disease in Eastern Mediterranean and Middle Eastern populations. Sjögren syndrome (8.9%) and other connective tissue diseases collectively accounted for a substantial proportion of diagnoses, highlighting the importance of rheumatology expertise in the management of systemic autoimmune conditions.

The comparison between newly diagnosed and undiagnosed patients showed significant differences. Newly diagnosed patients were significantly more likely to present with objective inflammatory features—including arthritis, elevated APR, Raynaud's phenomenon, dry mouth, ocular pathology, abdominal pain, and cytopenia—compared to those who remained undiagnosed. These findings are clinically intuitive: the presence of objective signs of systemic inflammation or organ involvement substantially increases the pre-test probability of an underlying rheumatic disease.^8^

Particularly noteworthy is the high prevalence of Raynaud’s phenomenon (20.0%) and abdominal pain (13.3%) in the newly diagnosed group. Raynaud’s phenomenon is a well-recognized early manifestation of connective tissue diseases, including systemic sclerosis, mixed connective tissue disease, and SLE, and its presence may warrant thorough rheumatologic evaluation including ANA testing and nailfold capillaroscopy. Abdominal pain in the context of a new rheumatologic diagnosis may reflect conditions such as Familial Mediterranean Fever (FMF), Behçet disease, or vasculitis—all of which are relatively prevalent in the Turkish population and may present with recurrent serositis or gastrointestinal involvement. Conversely, symptoms such as skin findings, RF positivity, ANA positivity, oral aphthae, and interstitial lung disease did not significantly differentiate the 2 groups in the cohort. The lack of significance for ANA positivity is particularly interesting, as ANA is frequently ordered as a screening test for connective tissue diseases. This finding may reflect the high rate of low-titre ANA positivity in the general population, which can lead to unnecessary rheumatology referrals and dilute the diagnostic specificity of this test in an outpatient setting.

The findings of this study have direct implications for the optimization of rheumatology subspecialty resources in Türkiye. First, the high proportion of follow-up visits suggests that structured protocols for stable patient management—potentially involving nurse practitioners, pharmacist-led monitoring, or telemedicine platforms—could substantially reduce the burden on rheumatologist time and improve access for new referrals. Second, the identification of symptom clusters associated with new diagnoses (particularly objective inflammatory features, Raynaud’s phenomenon, and abdominal pain) may generate hypotheses for the future development of evidence-based referral criteria for primary care providers, improving the efficiency of the referral pathway and reducing unnecessary consultations. Third, the diagnostic diversity observed in the cohort underscores the need for broad rheumatologic training and the maintenance of subspecialty expertise across the full spectrum of rheumatic diseases.

From a broader health system perspective, the data highlight the need for investment in rheumatology workforce development in Türkiye. With a rheumatologist density of approximately 0.22 per 100000 population—far below the levels observed in Western Europe and North America—the current workforce is insufficient to meet the growing demand for rheumatologic care. Strategies to address this shortage, as outlined by a recent review, include expanding fellowship training programs, integrating advanced practice providers into rheumatology teams, and leveraging telemedicine to extend the reach of specialist care to underserved regions.^9^

This study has several limitations that should be acknowledged. First, the single-center design limits the generalizability of the findings to other rheumatology settings in Türkiye, which may differ in terms of patient demographics, referral patterns, and diagnostic capacity. Second, the 2-month study period, while sufficient to capture a representative cross-section of clinic activity, may not fully reflect seasonal variation in disease presentation or referral patterns. Third, the study was conducted at a tertiary hospital in Türkiye, which may attract a more complex patient population than community-based rheumatology practices. Fourth, the categorization of patients as “undiagnosed” reflects the absence of a rheumatologic diagnosis at the time of the study visit; some of these patients may subsequently receive a diagnosis following further investigation or clinical evolution. Fifth, the relatively small number of newly diagnosed patients (n = 45) limits the statistical power of subgroup analyses. Finally, the relatively small number of newly diagnosed patients and the low frequency of several clinically relevant variables limited the feasibility of robust multivariable logistic regression analysis; therefore, the comparative findings should be interpreted as exploratory and hypothesis-generating.

This prospective study provides a detailed characterization of the patient population attending a tertiary rheumatology outpatient clinic in Türkiye over a 2-month period. The predominance of follow-up visits, the high diagnostic yield among new referrals, and the identification of clinical features associated with new rheumatologic diagnoses collectively highlight both the strengths and the challenges of current rheumatology practice in Türkiye. Peripheral joint pain remains the dominant presenting complaint, and over half of all patients carry an established rheumatologic diagnosis. The presence of objective inflammatory features—particularly arthritis, elevated APR, Raynaud’s phenomenon, and abdominal pain—was significantly associated with a new rheumatologic diagnosis in this cohort, suggesting that these features may be useful in guiding triage decisions; however, prospective validation in larger, multicentre studies is needed before formal referral recommendations can be made. These findings underscore the need for structured approaches to subspecialty resource optimization, including enhanced primary care education, evidence-based referral criteria, and innovative care delivery models, to ensure that the limited rheumatology workforce in Türkiye is deployed as effectively as possible.

## Figures and Tables

**Table 1.** Demographic and General Characteristics of the Study Population

Age, years, median [IQR]	53 [41-62]
Age group, n (%)	
18-29 years	99 (10.2)
30-44 years	208 (21.4)
45-59 years	343 (35.3)
60-74 years	276 (28.4)
75+ years	45 (4.6)
Sex, n (%)	
Female	719 (74.0)
Male	252 (26.0)
Visit type, n (%)	
Follow-up patient	491 (50.6)
Newly diagnosed	45 (4.6)
Other/first visit	435 (44.8)
Total patients with rheumatologic diagnosis, n (%)	536 (55.2)

Continuous variables are presented as median [IQR]. Nominal variables are presented as frequency (%).

**Table 2.** Symptoms Presented by Patients in the Undiagnosed Rheumatology Outpatient Clinic (n = 480)

Symptom/Finding	n	%
Peripheral joint pain	268	55.8
Low back pain	38	7.9
Arthritis	37	7.7
Myalgia	36	7.5
Dorsalgia	13	2.7
Ocular findings (uveitis, episcleritis, and dry eye)	13	2.7
Dry mouth	10	2.1
Skin redness and/or rash	10	2.1
Raynaud’s phenomenon	10	2.1
Abdominal pain	8	1.7
Recurrent oral aphthous ulcers	7	1.5
Neck pain	6	1.3
Interstitial lung disease	6	1.3
Allergic symptoms	5	1.0
Cytopenia	5	1.0
Thrombosis	3	0.6
Proteinuria	2	0.4
Erythema nodosum	1	0.2
Alopecia	1	0.2
Pericardial effusion	1	0.2

**Table 3.** Distribution of Rheumatologic Diagnoses Among Patients with a Rheumatologic Diagnosis: All Diagnosed Patients (n = 536) vs. Follow-up Patients (n = 491)

	n	%	n	%
Inflammatory arthritis				
Rheumatoid arthritis	163	30.4	147	29.9
Ankylosing spondylitis	75	13.9	70	14.2
Psoriatic arthritis	37	6.9	34	6.9
Undifferantiated spondyloarthropathy	11	2	8	1.6
Reactive arthritis	3	0.5	2	0.4
Juvenile idiopathic arthritis	2	0.3	2	0.4
Autoinflammatory/crystal arthropathies				
Familial Mediterranean fever	70	13	66	13.4
Behçet disease	23	4.3	21	4.3
Gout	45	8.4	42	8.6
Pseudogout	4	0.7	3	0.6
Connective tissue diseases				
Systemic lupus erythematosus	13	2.4	12	2.4
Sjögren syndrome	48	8.9	45	9.2
Systemic sclerosis	9	1.7	9	1.8
Undifferentiated CTD	10	1.8	8	1.6
Vasculitis and vascular diseases/other				
Vasculitis and vascular diseases, other	12	2.2	12	2.4
Polymyalgia rheumatica	8	1.5	6	1.2
Sarcoidosis	4	0.7	4	0.8

Nominal variables are presented as frequency (n) and percentage (%). Percentages are calculated over the respective group totals (n = 536 and n = 491). Some patients may have more than one diagnosis.

CTD, connective tissue disease.

**Table 4.** Comparison of Symptoms and Findings According to Rheumatologic Diagnosis

Variable	New Rheumatologic Diagnosis (n = 45)	No Diagnosis (n = 435)	*P*
Age, years—median [IQR]	51 [42-60]	53 [40-62]	0.639
Female sex	37 (82.2)	311 (71.5)	0.174
Peripheral joint pain	**33 (73.3)**	**235 (54.0)**	**0.020**
Back pain	**8 (17.8)**	**30 (6.9)**	**0.022**
Myalgia	**18 (40.0)**	**18 (4.1)**	**<0.001**
Arthritis	**19 (42.2)**	**18 (4.1)**	**<0.001**
ANA positivity	4 (8.9)	46 (10.6)	0.923
Ocular pathology	**6 (13.3)**	**7 (1.6)**	**<0.001**
Elevated APR	**11 (24.4)**	**13 (3.0)**	**<0.001**
Skin findings	1 (2.2)	9 (2.1)	1.000
RF positivity	3 (6.7)	22 (5.1)	0.912
Oral aphthae	2 (4.4)	5 (1.1)	0.270
Dry mouth	**5 (11.1)**	**5 (1.1)**	**<0.001**
Raynaud phenomenon	**9 (20.0)**	**1 (0.2)**	**<0.001**
Art./ven. thrombosis	0 (0.0)	4 (0.9)	1.000
Abdominal pain	**6 (13.3)**	**2 (0.5)**	**<0.001**
Cytopenia	**4 (8.9)**	**1 (0.2)**	**<0.001**
Proteinuria	1 (2.2)	1 (0.2)	0.179
History of miscarriage	0 (0.0)	7 (1.6)	0.838
Interstitial lung disease	2 (4.4)	4 (0.9)	0.186

Continuous variables are presented as median [IQR]. Nominal variables are presented as frequency (%). Chi-square test was used for categorical variables; Mann–Whitney *U-*test was used for continuous variables. Bold values indicate statistical significance (*P* < .05).

ANA, antinuclear antibody; APR, acute phase reactants; RF, rheumatoid factor.

## Data Availability

The data that support the findings of this study are available on request from the corresponding author.
